# Usefulness of C_2_HEST Score in Predicting Clinical Outcomes of COVID-19 in Heart Failure and Non-Heart-Failure Cohorts

**DOI:** 10.3390/jcm11123495

**Published:** 2022-06-17

**Authors:** Piotr Rola, Adrian Doroszko, Małgorzata Trocha, Katarzyna Giniewicz, Krzysztof Kujawa, Jakub Gawryś, Tomasz Matys, Damian Gajecki, Marcin Madziarski, Stanisław Zieliński, Tomasz Skalec, Jarosław Drobnik, Agata Sebastian, Anna Zubkiewicz-Zarębska, Barbara Adamik, Krzysztof Kaliszewski, Katarzyna Kiliś-Pstrusinska, Agnieszka Matera-Witkiewicz, Michał Pomorski, Marcin Protasiewicz, Janusz Sokołowski, Szymon Włodarczak, Ewa Anita Jankowska, Katarzyna Madziarska

**Affiliations:** 1 Department of Cardiology, Provincial Specialized Hospital, Iwaszkiewicza Street 5, 59-220 Legnica, Poland; 2 Clinical Department of Internal Medicine, Hypertension and Clinical Oncology, Wroclaw Medical University, Borowska 213, 50-556 Wroclaw, Poland; adrian.doroszko@umw.edu.pl (A.D.); jakub.gawrys@umw.edu.pl (J.G.); tomasz.matys@umw.edu.pl (T.M.); damian.gajecki@umw.edu.pl (D.G.); 3 Department of Pharmacology, Wroclaw Medical University, Mikulicz-Radecki Street 2, 50-345 Wroclaw, Poland; malgorzata.trocha@umw.edu.pl; 4 Statistical Analysis Centre, Wroclaw Medical University, K. Marcinkowski Street 2-6, 50-368 Wroclaw, Poland; katarzyna.giniewicz@umw.edu.pl (K.G.); krzysztof.kujawa@umw.edu.pl (K.K.); 5 Clinical Department of Rheumatology and Internal Medicine, Wroclaw Medical University, Borowska Street 213, 50-556 Wroclaw, Poland; madziarski.marcin@gmail.com (M.M.); agata.sebastian@umw.edu.pl (A.S.); 6 Clinical Department of Anaesthesiology and Intensive Therapy, Wroclaw Medical University, Borowska Street 213, 50-556 Wroclaw, Poland; stanislaw.zielinski@umw.edu.pl (S.Z.); tomasz.skalec@umw.edu.pl (T.S.); barbara.adamik@umw.edu.pl (B.A.); 7 Department of Population Health, Division Epidemiology and Health Education, Wroclaw Medical University, Bujwida Street 44, 50-368 Wroclaw, Poland; jaroslaw.drobnik@umw.edu.pl; 8 Clinical Department of Gastroenterology and Hepatology, Wroclaw Medical University, Borowska Street 213, 50-556 Wroclaw, Poland; anna.zubkiewicz-zarebska@umw.edu.pl; 9 Clinical Department of General, Minimally Invasive and Endocrine Surgery, Wroclaw Medical University, Borowska Street 213, 50-556 Wroclaw, Poland; krzysztof.kaliszewski@umw.edu.pl; 10 Clinical Department of Paediatric Nephrology, Wroclaw Medical University, Borowska Street 213, 50-556 Wroclaw, Poland; katarzyna.kilis-pstrusinska@umw.edu.pl; 11 Screening of Biological Activity Assays and Collection of Biological Material Laboratory, Wroclaw Medical University Biobank, Wroclaw Medical University, Borowska Street 211A, 50-556 Wroclaw, Poland; agnieszka.matera-witkiewicz@umw.edu.pl; 12 Clinical Department of Gynecology and Obstetrics, Wroclaw Medical University, Borowska Street 213, 50-556 Wroclaw, Poland; michal.pomorski@umw.edu.pl; 13 Institute of Heart Diseases, Wroclaw Medical University, Borowska Street 213, 50-556 Wroclaw, Poland; marcin.protasiewicz@umw.edu.pl (M.P.); ewa.jankowska@umw.edu.pl (E.A.J.); 14 Institute of Heart Diseases, University Hospital, Borowska Street 213, 50-556 Wroclaw, Poland; 15 Clinical Department of Emergency Medicine, Wroclaw Medical University, Borowska Street 213, 50-556 Wroclaw, Poland; janusz.sokolowski@umw.edu.pl; 16 Department of Cardiology, The Copper Health Centre (MCZ), 59-300 Lubin, Poland; wlodarczak.szy@gmail.com; 17 Clinical Department of Nephrology and Transplantation Medicine, Wroclaw Medical University, Borowska Street 213, 50-556 Wroclaw, Poland; katarzyna.madziarska@umw.edu.pl

**Keywords:** heart failure, COVID-19, SARS-CoV-2, outcomes, C_2_HEST score, mortality, prediction

## Abstract

Background: Patients with heart failure represent a vulnerable population for COVID-19 and are prone to having worse prognoses and higher fatality rates. Still, the clinical course of the infection is dynamic, and complication occurrence in particular in patients with heart failure is fairly unpredictable. Considering that individual components of the C_2_HEST (C2: Coronary Artery Diseases (CAD)/Chronic obstructive pulmonary disease (COPD); H: Hypertension; E: Elderly (Age ≥ 75); S: Systolic HF; T: Thyroid disease) are parallel to COVID-19 mortality risk factors, we evaluate the predictive value of C_2_HEST score in patients with heart failure (HF) Material and Methods: The retrospective medical data analysis of 2184 COVID-19 patients hospitalized in the University Hospital in Wroclaw between February 2020 and June 2021 was the basis of the study. The measured outcomes included: in-hospital mortality, 3-month and 6-month all-cause-mortality, non-fatal end of hospitalization, and adverse in-hospital clinical events. Results: The heart failure cohort consists of 255 patients, while 1929 patients were assigned to the non-HF cohort. The in-hospital, 3-month, and 6-month mortality rates were highest in the HF cohort high-risk C_2_HEST stratum, reaching 38.61%, 53.96%, and 65.36%, respectively. In the non-HF cohort, in-hospital, 3-month, and 6-month mortalities were also highest in the high-risk C_2_HEST stratum and came to 26.39%, 52.78%, and 65.0%, respectively. An additional point in the C_2_HEST score increased the total death intensity in 10% of HF subjects (HR 1.100, 95% CI 0.968–1.250 *p* = 0.143) while in the non-HF cohort, the same value increased by 62.3% (HR 1.623, 95% CI 1.518–1.734 *p* < 0.0001). Conclusions: The C_2_HEST score risk in the HF cohort failed to show discriminatory performance in terms of mortality and other clinical adverse outcomes during hospitalization. C_2_HEST score in the non-HF cohort showed significantly better performance in terms of predicting in-hospital and 6-month mortality and other non-fatal clinical outcomes such as cardiovascular events (myocardial injury, acute heart failure, myocardial infarction, cardiogenic shock), pneumonia, sepsis, and acute renal injury.

## 1. Introduction

Since the outbreak of the global COVID-19 pandemic, numerous risk factors of SARS-CoV-2 infection severity have been described [1,2]. Even though several scoring systems [3] have been proposed, the clinical course among individuals with COVID-19 is still unpredictable. Therefore, the relevant need for a more accurate evaluation of potential outcomes to support clinical decisions and, consequently, to avoid unnecessary resource consumption is a crucial point in preventing public health collapse.

Patients with congestive heart failure due to advanced age and coexisting comorbidities constitute a particularly challenging subpopulation experiencing worse outcomes, including increased mortality. In addition, in the course of SARS-CoV-2 infection, they face low cardiovascular reserve, increased metabolic demand, uncontrolled immune response [4], and thromboembolic issues [5]. Due to limited resources, all additional diagnostic and prognostic features seem to be advisable to support life-saving interventions and improve decision-making processes regarding hospital admission and an inpatient referral.

Originally, the C_2_HEST score was proposed as a scoring system to allow the stratification of the risk of developing atrial fibrillation (AF) in the general population [6]. However, recently, Liang et al. [7] demonstrated that C_2_HEST score could also predict adverse clinical outcomes, including death and hospitalization, among patients with heart failure. Considering that individual components of the C_2_HEST (C2: CAD/COPD; H: Hypertension; E: Elderly (Age ≥ 75); S: Systolic HF; T: Thyroid disease) are parallel to COVID-19 mortality risk factors, juxtaposing it with the outcomes of recently published studies [8,9,10] suggesting that this simple scoring system has the ability to predict outcomes in several COVID-19 subpopulations, we designed this study to evaluate the predictive value of C_2_HEST score in patients with heart failure (HF).

## 2. Materials and Methods

### 2.1. Study Population

The study population consisted of 2184 patients hospitalized in the University Hospital in Wroclaw between February 2020 and June 2021 with confirmed infection of SARS-CoV-2 virus (diagnosis was based on results of reverse transcription-polymerase chain reaction (RT-PCR) for viral RNA from nasopharyngeal swab specimens). The study (COronavirus in Lower Silesia (COLOS) study) had the approval of the local ethics committee (No: KB-444/2021). Due to the retrospective, observational character of the study, written informed consent for participation was waived.

Anonymized medical data of all study subjects were preanalyzed and assigned to one of the two study cohorts. For the first study arm, we incorporated patients with a history of HF diagnosed prior to index hospitalization. Diagnosis of heart failure was based on the European Society of Cardiology (ESC) guidelines [11]. The second study arm was composed of patients without a previous diagnosis of HF.

For all patients recruited to both study cohorts, we evaluated the C_2_HEST value. C_2_HEST score analysis was performed based on original variables; coronary artery disease (1 point), chronic obstructive pulmonary disease (1 point), hypertension (1 point), elderly (age ≥ 75 years, 2 points), systolic HF (2 points), and thyroid disease (1 point). Depending on the calculated score, subjects were allocated to one of three strata: low-risk, 0 or 1 point; medium-risk, 2 or 3 points; and high-risk, 4 or more points.

### 2.2. Endpoints and Outcomes

The primary clinical outcome was defined as in-hospital, 3-month, and 6-month all-cause mortality. Additionally, data regarding other clinical outcomes focused on the in-hospital period were collected. The measured outcomes included: end of hospitalization other than death (discharge, deterioration, or recovery with transfer to another hospital), level of respiratory support, shock, sepsis, systemic inflammatory response syndrome (SIRS), acute kidney injury, acute liver dysfunction, pneumonia, myocardial injury, acute heart failure, stroke, pulmonary embolism, and all-type symptomatic bleeding.

### 2.3. Statistical Analysis

R language version 4.0.4 with additional packages [12,13,14,15] pROC and time-ROC, coin, survival, and odds ratio was used by professional academic statisticians to perform data analysis. A significance value level was set at 0.05. Descriptive data regarding categorical variables were presented as numbers and percentages, while numerical variables were presented as mean with standard deviation, range (minimum–maximum), and the number of non-missing values. Chi-square and Omnibus tests were used in the case of categorical variables which exceeded 5 expected cases in each group. The Fisher exact test was used for subjects with fewer cell counts. Welch’s ANOVA was performed to analyze continuous variables to adjust for unequal variances among the risk-strata and a sample size large sufficient for the appropriateness of asymptotic results. In the case of continuous variables, the Games–Howell variant of Tukey correction was used as a part of a post hoc analysis. The post hoc test, for categorical variables, was analogous to the omnibus test. Therefore, it was used in subgroups with a Bonferroni correction. In-hospital mortality along with all-cause mortality were right-censored data. Therefore, time-dependent ROC analysis with inverse probability of censoring weighting (IPCW) was used to estimate them. The time-dependent area under the curve (AUC) was used to assess the C_2_HEST score. The log-rank test was used as a part of the confirmation of differences in survival curves among risk strata.

The Grambsch–Therneau test was used to verify proportional hazard assumption. The Cox proportional hazard model was used to perform an analysis of the hazard ratio (HR) in the C_2_HEST score, its components, and the risk strata. The logistic regression model was used during an analysis of secondary outcomes due to their dichotomic nature. Classical ROC analysis with an AUC measure was performed to assess predictive capability. Odds ratio (OR) was presented as a size effect for the influence of the C_2_HEST score, its components, and risk strata.

## 3. Results

### 3.1. Baseline Demographical and Clinical Features of the Studied Population

The baseline demographic characteristics, along with a past medical history of subjects allocated to both study cohorts, were presented in Table 1. The heart failure group consisted of 255 mainly male patients (144 (56.47%)). In this study cohort, no subjects were allocated to the low-risk stratum. This fact is related to the fundamentals of the C_2_HEST score—the previous diagnosis of systolic HF results in increasing the value of a score up to 2 points and automatically allocates all patients with HF to the medium- or high-risk strata. The vast majority of patients in the HF arm were assigned to the high-risk stratum (*n*= 202), while the medium-risk stratum consisted of 53 patients. In total, 1929 patients were assigned to the non-HF cohort. Among all C_2_HEST risk strata in the study, the most numerous was the low-risk non-HF, with 1417 participants; medium-risk consisted of 439 subjects, and high-risk consisted of 72 patients.

In both study cohorts, an increase in the C_2_HEST risk stratum resulted in more advanced subject age and a higher prevalence of comorbidities. Differences between study cohorts were regarding the prevalence of diabetes, atrial fibrillation, valvular heart disease, peripheral artery disease, stroke/TIA, and chronic kidney disease.

In particular, the non-HF cohort revealed significant differences between C_2_HEST risk groups in terms of treatment applied before admission to the hospital. Detailed data are presented in Supplementary Table S1.

Analysis of baseline patient-reported symptoms and vital signs (Table 2) revealed significant differences among prevalence, systolic blood pressure, oxygen saturation (SpO_2_) in room air, crackles, wheezing, pulmonary congestion, and peripheral oedema in the non-HF cohort, whereas no differences were observed in the HF cohort.

The detailed characteristics of the laboratory parameters measured during admission and discharge from the hospital in both study cohorts are pooled in Supplementary Table S2. At the time of admission, the decrease in hemoglobin levels correlated with the increase of risk in C_2_HEST score in both study arms. Interestingly, there were no significant differences between risk groups in HF and non-HF arms in terms of arterial blood gases (ABG) and acid–base balance parameters nor in the admission level of inflammatory markers (leucocytes, CRP, procalcitonin, IL-6). At admission, the non-HF high-risk stratum was characterized by more pronounced laboratory features of renal failure (higher level of creatinine and urea, along with lower eGFR). Additionally, in the non-HF cohort, admission levels of cardiac injury markers (TnI and NT-proBNP) rose together with the level of risk in the C_2_HEST score.

### 3.2. Treatment Applied during Hospitalization

All differences in applied therapy during the hospitalization period between the C_2_HEST group among both study cohorts are pooled in Supplementary Table S3. Subjects in non-HF cohorts in the higher C_2_HEST stratum were prone to receiving antimicrobial treatment. On the other hand, convalescent plasma was less frequently used in this subpopulation of patients. No statistically significant differences in applied therapy between all risk strata were observed in the HF cohort.

No significant differences in respiratory support were observed in the HF cohort. In the non-HF cohort, the assignment to a specific C_2_HEST stratum score correlated with the advancement of respiratory support applied during the hospitalization C_2_HEST. Along with increasing C_2_HEST score value, prevalence of noninvasive ventilation increased, whereas the oxygenation parameters from the period of qualification for advanced respiratory support decreased. On the other hand, the frequency of implementation of invasive ventilation decreased in parallel with the rise in C_2_HEST score value. No differences in either study cohort were shown regarding the need for urgent coronary revascularization procedures during the index hospitalization, need for catecholamine administration, nor for use of hemodialysis (Table 3).

### 3.3. Association C_2_HEST Score with Results and Mortality

The *in-hospital*—and then the 3-*month* and 6-*month*—mortality rates were the highest in the *high-risk* HF cohort C_2_HEST stratum, reaching 65.36%, 53.96%, and 38.61%, respectively. Interestingly, in this study cohort, significant differences in mortality rates between the *medium-risk* stratum and *high-risk* stratum were observed only for in-hospital mortality. Regarding the post-discharge period, no similar relationship was observed. All data regarding short and long-term mortality were pooled in Table 4. In the non-HF cohort, *in-hospital*, *3-month*, and *6-month* mortality were also highest in the *high-risk* C_2_HEST stratum and came to 26.39%, 52.78%, and 65.0%, respectively. In this study, arm differences between *low-risk vs. high-risk* and low-*risk vs. medium-risk* C_2_HEST groups were statistically significant.

### 3.4. The All-Cause Mortality Discriminatory Performance of the C_2_HEST Score

**Figure 1:**
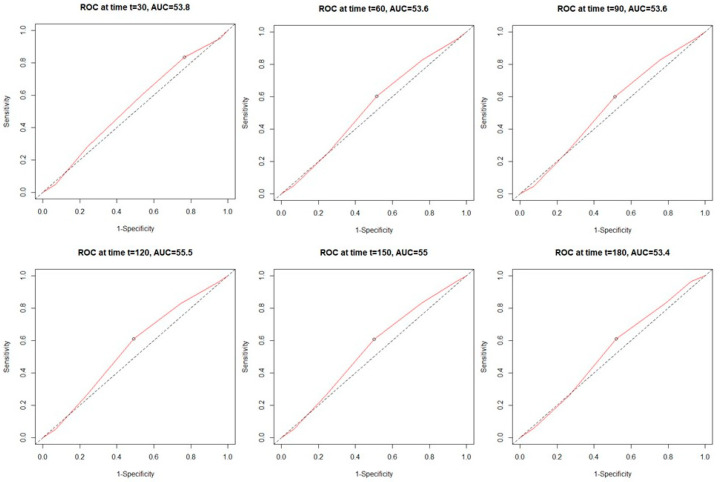
The time-dependent receiver operating characteristic (ROC) for all-cause mortality in the heart failure cohort.

**Figure 2:**
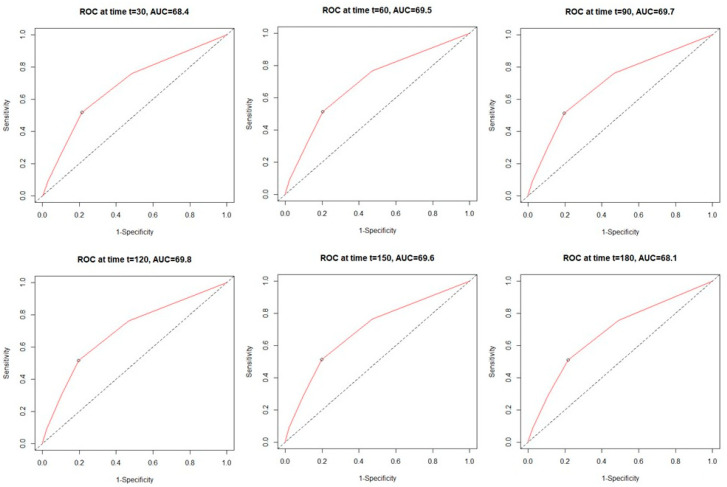
The time-dependent receiver operating characteristic (ROC) for all-cause mortality in the non-heart-failure cohort.

**Figure 3:**
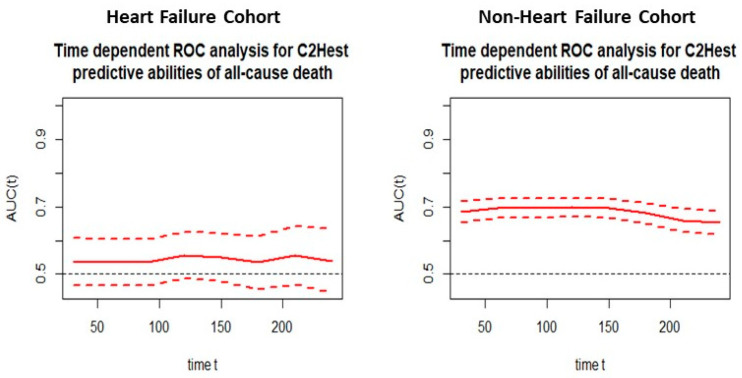
Time-dependent ROC analysis for the C_2_HEST predictive abilities of all-cause death in both study cohorts.

**Figure 4:**
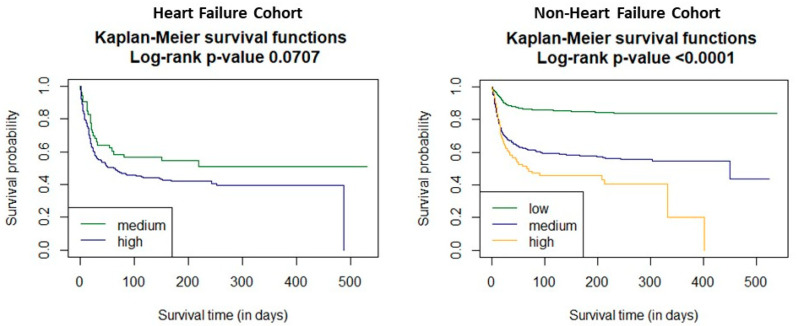
The survival curves for the C_2_HEST strata in both study cohorts, estimated by Kaplan–Meier function.

Analysis of the time-dependent receiver operating characteristic (ROC) in both study cohorts revealed higher sensitivity of the C_2_HEST scale in the non-HF cohort compared to HF subjects. (Figure 1 and Figure 2). C_2_HEST predicting AUC in the HF cohort failed to predict all-cause mortality [16]. In the non-HF cohort, the AUC value was significantly higher. Analysis of the ACU values of the HF cohort vs. the non-HF cohort in different periods revealed that at 1-month, AUC = 53.8 vs. 68.4%; 3-month AUC = 53.6 vs. 69.7%; 6-month AUC = 53.4 vs. 68.1%. The “all-cause death” time-dependent AUC for the C_2_HEST score was presented in Figure 3. Additionally, Kaplan–Meier survival curves for the C_2_HEST strata were presented in Figure 4. The *p* value for the log-rank test was <0.0001. We have observed differences in estimated survival probability in both study cohorts. Practically, starting from admission time, subjects in the HF cohorts were less likely to survive COVID-19 compared to the non-HF cohort.

We performed Cox model analysis regarding the predictive value of C_2_HEST score in terms of mortality in both study cohorts. In the overall model with uncategorized value of C_2_HEST, additional points in the C_2_HEST score were related to an increase of the total death intensity in 10% of HF subjects, while in the non-HF subpopulation, that value was 62.3% (Table 5 and Table 6). In the categorized model, the change from the medium to the high category in the heart failure cohort increased death expectation by 46.5%. On the other hand, transfer from the low-risk to the medium-risk stratum increased the all-cause death intensity by three times, while the shift from low-risk to high-risk increased the probability of death by almost five times (Table 5 and Table 6).

The associations of individual C_2_HEST score components with mortality in both study cohorts are presented in Table 7 and Table 8. The highest prognostic value for all-cause death in both study groups was noticed for age (1.4743 in HF subjects vs. 3.211 in men). Interestingly, among all individual C_2_HEST score components in the HF cohort, only age over 75 and thyroid diseases were associated with a significant change in HR for death. However, in the non-HF cohort, CAD, COPD, age over 75, and hypertension increased the HR for death.

In order to assess whether the original cut-off values of C_2_HEST score risk (the low/medium/high-risk categories for 0–1/2–3/ ≥ 4 points, respectively) are the best possible stratification system for both study cohorts, we evaluated the difference in Kaplan–Meier survival curves, and all the possible C_2_HEST intervals were analyzed. Additionally, the log-rank statistics was calculated (Supplementary Tables S4 and S5). In terms of the HF cohort categories, the values of 0–3/4–6/7–8 points (the low/medium/high-risk categories, respectively) were revealed to be characterized by better separation than was generally accepted. Meanwhile, for non-HF cohort values, 0/1/2–8 points (the low/medium/high-risk categories, respectively) showed the highest separation accuracy.

### 3.5. Association of C_2_HEST Score with Non-Fatal Outcomes

**Figure 5:**
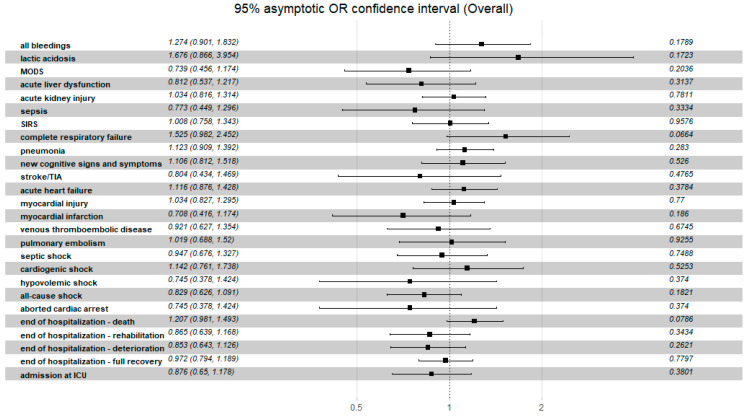
The overall odds ratio for the discriminatory performance of the C_2_HEST score on clinical non-fatal events in the HF cohort. Abbreviations: MODS—multiple organ dysfunction syndrome, TIA—transient ischemic attack, SIRS—systemic inflammatory response syndrome.

**Figure 6:**
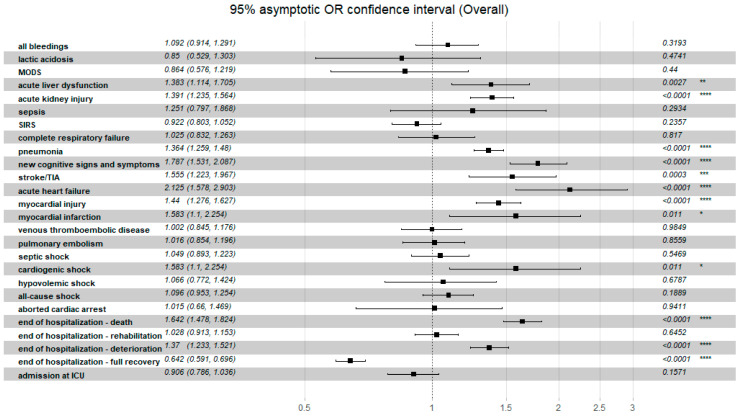
The overall odds ratio for the discriminatory performance of the C_2_HEST score on clinical non-fatal events in the non-HF cohort. Abbreviations: MODS—multiple organ dysfunction syndrome, TIA—transient ischemic attack, SIRS—systemic inflammatory response syndrome. Significance code: * < 0.05; ** < 0.01; *** < 0.001; **** < 0.0001.

All the data regarding the relationship of clinical non-fatal events and the C_2_HEST risk strata in both study cohorts are presented in the Table 9 and Figure 5 and Figure 6. In the heart failure cohort, none of the study’s secondary endpoints showed significant differences in prevalence among the original C_2_HEST score risk strata. In the non-HF cohort assignment to C_2_HEST, higher risk strata were correlated with a higher probability of clinical deterioration (transfer to another hospital) and lower probability for full recovery (discharge to home). Additionally, subjects with higher C_2_HEST score values were more prone to experience some cardiovascular complications (including myocardial injury, acute heart failure episode, stroke/TIA) during a hospitalization period. Moreover, high C_2_HEST scores in the non-HF subpopulation were associated with a higher probability of pneumonia, sepsis, and acute kidney injury.

## 4. Discussion

Patients with cardiovascular disease represent a vulnerable population for worse COVID-19 outcomes [17,18,19]. Considering that chronic heart failure (CHF) in modern societies involves more than 2% of the general population [20] and approximately 10% of the population over 70, a similarly high number is highly susceptible to unfavorable COVID-19 outcomes, including high hospitalization rate, longer duration of in-hospital treatment, ICU admission rate, and higher mortality rate. Even so, in these high-risk subjects, the clinical course of the infection remains dynamic and hardly predictable, particularly at the time of admission. Additionally, overlapping the clinical and radiological presentations provides additional difficulties in the correct triage of patients admitted to the hospital.

Accurately performed risk stratification in individual patients can provide adequate guidance, allowing for reasonable management of limited resources during a COVID-19 pandemic. Unfortunately, a simple, fast, well-validated scoring system dedicated to the HF subpopulation is still missing. Taking into account some encouraging data from our previous analyses concerning other subpopulations of COVID-19 patients [8,9,19], we decided to validate the C_2_HEST score system in terms of heart failure subjects.

According to the theoretical assumptions, the C_2_HEST score scale is well correlated with patient comorbidity rate in both study cohorts, but this relationship was more pronounced in the HF group and predicted a wide spectrum of cardiovascular disorders (Table 1). Not surprisingly, a reflection of this finding was the prevalence of specific, targeted pre-hospital treatment (ACEI, ARBN, β-blocker, diuretics, statins) applied in the HF cohort [20]. This relationship was not present in the non-HF cohort. Nevertheless, this therapy was associated with a reduction in mortality and rehospitalization in patients with cardiovascular disease, particularly those with heart failure and coronary artery disease. It is worth noting that in patients with active SARS-CoV-2 infections, some safety concerns are still rising, and an intense debate is ongoing on this matter [21,22,23]. Some recently published large-population studies on COVID-19 [22,24] have proven that the number of cardiovascular comorbidities appears to be independently associated with increased COVID-19-related death. It is worth noting that no relationship between commonly used CVD medications and increased risk of death due to COVID-19 was identified. Furthermore, some data suggest that specific cardiovascular drugs should be continued in order to reduce potential unfavorable cardiovascular events in the course of SARS-CoV-2 infection, particularly in subjects with heart failure.

In contrast to the earlier reports [25] suggesting that increased inflammatory response with subsequent increased production of inflammatory markers in patients with HF could potentially affect the coagulation cascade, induce an endothelial dysfunction, and hemodynamic imbalance [26,27] leading to decompensation of heart failure, thus increasing the rate of an unfavorable outcome [28], in our cohort study, no significant differences were observed regarding the levels of inflammatory markers on admission. A similar interesting observation was made for the non-HF cohort. Surprisingly, in the HF cohort, there were no differences between C_2_HEST score risk strata in terms of need for use of ventilation support during hospitalization. However, at the same time, in the non-HF cohort, we observed an increase in respiratory support parallel to the coexisting increase in the C_2_HEST score risk category.

In our study in the HF cohort, the C_2_HEST score risk failed to show discriminatory performance in terms of mortality (Figure 1 and Figure 3). It is likely that HF by itself is a strong risk factor for poor COVID-19 outcomes when hospitalization was required. The additional discriminants from the C_2_HEST score scale did not allow for an appropriate selection of patients with higher overall mortality. This is partially confirmed by the high in-hospital mortality in the HF cohort—approximately 38% in high-risk C_2_HEST score. Those data are additionally visualized in Kaplan–Maier curves for the heart failure cohort (Figure 4). A similar observation was previously made in terms of other COVID-19 strong risk factors (diabetes and coronary artery diseases) [9]. On the other hand, in the non-HF cohort, we observed significantly better performance of C_2_HEST scores in terms of morality discriminatory ability. Furthermore, in the HF cohort, we observed the inability of C_2_HEST to predict other non-fatal secondary outcomes during the hospitalization period. Similarly, in the non-HF subjects, C_2_HEST scores were able to predict in-hospital cardiovascular complications (such as myocardial injury, acute heart failure episode, stroke/TIA) as well as pneumonia, acute kidney injury, and sepsis.

Interestingly, so far, several prognostic scales for COVID-19 have been introduced, including the COVID-Gram Risk Score, the PRIEST score [29], and the Brescia COVID Severity Scale (BRCSS) [3]. Nevertheless, mostly due to their complexity (assessment based on laboratory assays, clinical data, and radiographic imaging), their implementation into everyday clinical practice as a routine triage tool is limited. Therefore, the data obtained in our study suggest that the C_2_HEST score is a useful triage tool that could be used in the general population and not only in strictly selected high-risk populations.

A growing body of evidence indicates that some diagnostic tools, including the lung ultrasound (LUC) may present some usefulness in diagnosis, optimization of treatment, and risk stratification in COVID-19 patients [30,31,32]. Therefore, a multidimensional assessment of risk factors for an unfavorable outcome of COVID-19, including the data from imaging diagnostics, could constitute an interesting approach. Combining the C2HEST risk score with LUC might be valuable and increase the discriminatory performance of the C2HEST score in predicting outcomes without an unnecessary increase in the complexity of scale. Nevertheless, further studies evaluating the value of such a modified C2HEST score scale are necessary.

## 5. Limitations

This study contains several limitations, including the study design—particularly its retrospective, single-center, non-randomized character. The homogeneous study population focused on hospitalized subjects without ambulatory subjects. Additionally, all hospitalizations were carried out in extraordinary circumstances—during the global COVID-19 pandemic. Therefore, the clinical outcomes were probably partially affected by limited resources. Additionally, the study protocol did not include a routine in-hospital assessment of the LVEF (mainly due to safety concern), and the TTE was performed only in deteriorating/decompensating subjects when needed. Therefore, the allocation to the HF cohort was made on the basis of past medical history—the diagnosis of HFrEF or HFmrEF was made based on the TTE performed prior to admission to the hospital. Therefore, we decided not to present the data collected during hospitalization based mostly on the deteriorating subjects, as they could not reflect the whole studied cohort.

## 6. Conclusions

The present study is the first to demonstrate the differences in the predictive value of the C_2_HEST score between patients with and without heart failure hospitalized due to COVID-19. The C_2_HEST score risk in the HF cohort failed to show discriminatory performance in terms of mortality and other clinical adverse outcomes during hospitalization. On the other hand, in the non-HF cohort, it revealed significantly better performance in predicting in-hospital and 6-month-mortality as well as other non-fatal clinical outcomes, including not only cardiovascular events (myocardial injury, acute heart failure, myocardial infract, carcinogenic shock) but also pneumonia, sepsis, and acute renal injury. Therefore, C_2_HEST score, as a relatively simple and easy-to-apply tool, might become a useful tool for risk stratification in the general population, but not in the strictly selected high-risk subpopulation with congestive heart failure.

## Figures and Tables

**Table 1:** Baseline characteristics after C_2_HEST risk stratification in the HF and non-HF study cohorts.

Variables, Units (N) (HF/Non-HF)	Low Risk (0–1)		Medium Risk (2–3)		High Risk (≥4)		*t*-Test	OMNIBUS *p* Value	*p* Value for Post Hoc Analysis
	Min–Max (N) or n/N (% of Risk Category)		Min–Max (N) or n/N (% of Risk Category)		Min–Max (N) or n/N (% of Risk Category)				
	HF	Non-HF	HF	Non-HF	HF	Non-HF	HF	Non-HF	
	**Demographics**								
**Age, years** (255/1929)		51.11 ± 15.9 17–74 (1417)	63.38 ± 9.76 36–74 (53)	77.03 ± 11.1 29–100 (439)	77.71 ± 10.39 38–100 (202)	81.04 ± 4.93 73–94 (72)	<0.0001 ^c^	<0.0001	<0.0001 ^a,b,c^
**Male gender** (255/1929)		735/1417 (51.87%)	33/53 (62.26%)	175/439 (39.86%)	111/202 (54.95%)	28/72 (38.89%)	0.4236 ^c^	**<0.0001**	**<0.0001 ^a^** 0.1274 ^b^ **<0.0001 ^c^**
**BMI kg/m^2^** (69/485)		28.28 ± 5.07 15.36–49.38 (397)	29.36 ± 6.9 20.89–46.71 (17)	29.27 ± 5.36 18.59–47.75 (73)	28.12 ± 6.14 17.28–48.21 (52)	26.67 ± 4.71 16.41–34.89 (15)	0.5129 ^c^	0.146	N/A
**Obesity** **(BMI ≥ 30 kg/m^2^)** (69/485)		132/397 (33.25%)	8/17 (47.06%)	30/73 (41.1%)	17/52 (32.69%)	4/15 (26.67%)	0.7294 ^c^	0.3259	N/A
**Cigarette smoking** **never** **previous** **current** (255/1929)		1337/1417 (94.35%) 46/1417 (3.25%) 34/1417 (2.4%)	38/53 (71.7%) 8/53 (15.09%) 7/53 (13.29%)	393/439 (90.14%) 27/439 (6.19%) 16/439 (3.67%)	162/202 (80.2%) 24/202 (11.88%) 16/202 (7.92%)	56/72 (78.87%) 12/72 (16.9%) 3/72 (4.23%)	0.3396 ^c^	**<0.0001**	**0.0238 ^a^** **<0.0001 ^b^** **0.0334 ^c^**
**Comorbidities**									
**Hypertension** (255/1929)		415/1417 (29.29%)	36/53 (67.92%)	321/439 (73.12%)	179/202 (88.61%)	70/72 (97.22%)	**0.0005 ^c^**	**<0.0001**	**<0.0001 ^a,b,c^**
**DM** (255/1929)		208/1417 (14.68%)	20/53 (37.74%)	126/439 (28.77%)	94/202 (46.77%)	24/72 (33.39%)	0.4209 ^c^	**<0.0001**	**<0.0001 ^a^** **0.0028 ^b^** 0.879 ^c^
**Dyslipidemia** (172/653)		289/417 (69.3%)	26/34 (76.47%)	148/199 (74.37%)	117/138 (84.78%)	31/37 (83.78%)	0.3661 ^c^	0.1034	N/A
**Atrial fibrillation/flutter** (255/1929)		49/1417 (3.46%)	22/53 (41.51%)	84/439 (19.13%)	112/202 (55.45%)	23/72 (31.94%)	0.0982 ^c^	**<0.0001**	**<0.0001 ^a,b^** 0.061 ^c^
**Previous coronary revascularization** (255/1929)		6/1417 (0.42%)	4/53 (7.55%)	33/439 (7.52%)	89/202 (44.06%)	22/72 (30.56%)	**<0.0001 ^c^**	**<0.0001**	**<0.0001 ^a,b,c^**
**Previous myocardial infarction** (255/1929)		11/1417 (0.78%)	4/53 (7.55%)	59/439 (13.44%)	88/202 (43.56%)	29/72 (40.28%)	**<0.0001 ^c^**	**<0.0001**	**<0.0001 ^a,b,c^**
**Heart failure** (255/1929)		0/1417 (0%)	53/53 (100%)	0/439 (0%)	202/202 (100%)	0/72 (0%)	**<0.0001 ^c^**	**<0.0001**	**<0.0001 ^a,b,c^**
**Moderate/severe valvular heart disease** **or previous valve heart surgery** (255/1929)		13/1417 (0.92%)	16/53 (30.19%)	16/439 (3.64%)	48/202 (23.76%)	3/72 (4.17%)	0.434 ^c^	**0.0002**	**0.0007 ^a^** 0.1157 ^b^ 1.0 ^c^
**Peripheral artery disease** (255/1929)		26/1417 (1.83%)	6/53 (11.32%)	25/439 (5.69%)	37/202 (18.32%)	6/72 (8.33%)	0.3151 ^c^	**<0.0001**	**0.0002 ^a^** **0.0104 ^b^** 1.0 ^c^
**Previous stroke/TIA** (255/1929)		47/1417 (3.32%)	6/53 (11.32%)	53/439 (12.07%)	47/202 (23.27%)	11/72 (15.28%)	0.0859 ^c^	**<0.0001**	**<0.0001 ^a^** **0.0002 ^b^** 1.0 ^c^
**Chronic kidney disease** (255/1929)		70/1417 (4.94%)	14/53 (26.42%)	56/439 (12.76%)	78/202 (38.61%)	13/72 (18.06%)	0.1375 ^c^	**<0.0001**	**<0.0001 ^a,b^** 0.9041 ^c^
**Hemodialysis** (255/1929)		19/1417 (1.34%)	4/53 (7.55%)	16/439 (3.64%)	17/202 (8.42%)	2/72 (2.78%)	1.0 ^c^	**0.0078**	**0.0121 ^a^** 0.8097 ^b^ 1.0 ^c^
**Asthma** (255/1929)		54/1417 (3.81%)	3/53 (5.66%)	17/439 (3.87%)	7/202 (3.47%)	4/72 (5.56%)	0.4379 ^c^	0.6782	N/A
**COPD** (255/1929)		6/1417 (0.42%)	0/53 (0%)	25/439 (5.69%)	29/202 (14.36%)	15/72 (20.83%)	**0.0072 ^c^**	**<0.0001**	**<0.0001 ^a,b^** **0.0003 ^c^**
**Hypothyroidism** (255/1929)		76/1417 (5.36%)	0/53 (0%)	68/439 (15.49%)	33/202 (16.34%)	31/72 (43.06%)	**0.0004 ^c^**	**<0.0001**	**<0.0001 ^a,b,c^**
**Hyperthyroidism** (255/1929)		4/1417 (0.28%)	0/53 (0%)	10/439 (2.28%)	7/202 (3.47%)	0/72 (0%)			

Continuous variables are presented as mean ± SD, range (minimum–maximum), and number of non-missing values. Categorized variables are presented as a number with a percentage. Information about the numbers with valid values is provided in the left column. Abbreviations: CAD—coronary artery disease, OMNIBUS—analysis of variance, N—valid measurements, n—number of patients with parameter above cut-off point, SD—standard deviation, BMI—body mass index, DM—diabetes mellitus, TIA—transient ischemic attack, COPD—chronic obstructive pulmonary disease, N/A—not applicable, a—low risk vs. medium risk, b—low risk vs. high risk, c—medium risk vs. high risk. Bold text—statistically significant values.

**Table 2:** Patient-reported symptoms, vital signs, and abnormalities measured during physical examination at hospital admission in the HF and non-HF study cohorts after C_2_HEST risk stratification.

Variables, Units (N) (HF/Non-HF)	Low-Risk (0–1)		Medium-Risk (2–3)		High-Risk (≥4)		*t*-Test	OMNIBUS *p* Value	*p* Value for Post Hoc Analysis
	Min–Max (N) or n/N (% of Risk Category)		Min–Max (N) or n/N (% of Risk Category)		Min–Max (N) or n/N (% of Risk Category)				
	HF	Non-HF	HF	Non-HF	HF	Non-HF	HF	Non-HF	
**Patient-Reported Symptoms**									
**Cough** (255/1929)		455/1417 (31.11%)	13/53 (24.53%)	111/439 (25.28%)	54/202 (26.73%)	15/72 (20.83%)	0.8814 ^c^	**0.0053**	**0.0238 ^a^** 0.181 ^b^ 1.0 ^c^
**Dyspnea** (255/1929)		569/1417 (40.16%)	32/53 (60.38%)	174/439 (39.64%)	112/202 (55.45%)	34/72 (47.22%)	0.6249 ^c^	0.4661	N/A
**Chest pain** (255/1929)		102/1417 (7.2%)	8/53 (15.09%)	26/439 (5.92%)	21/202 (10.4%)	6/72 (8.33%)	0.4741 ^c^	0.5872	N/A
**Hemoptysis** (255/1929)		9/1417 (0.64%)	0/53 (0%)	2/439 (0.46%)	4/202 (1.98%)	0/72 (0%)	0.5831 ^c^	1.0	N/A
**Smell dysfunction** (255/1929)		61/1417 (4.3%)	1/53 (1.89%)	9/439 (2.05%)	3/202 (1.49%)	2/72 (2.78%)	1.0 ^c^	0.0731	N/A
**Taste dysfunction** (255/1929)		49/1417 (3.46%)	2/53 (3.77%)	8/439 (1.82%)	3/202 (1.49%)	4/72 (5.56%)	0.2782 ^c^	0.0851	N/A
**Abdominal pain** (255/1929)		103/1417 (7.27%)	2/53 (3.77%)	24/439 (5.47%)	14/202 (6.93%)	3/72 (4.17%)	0.5358 ^c^	0.3319	N/A
**Diarrhea** (255/1929)		75/1417 (5.3%)	2/53 (3.77%)	31/439 (7.06%)	14/202 (6.93%)	5/72 (6.94%)	0.5358 ^c^	0.325	N/A
**Nausea/Vomiting** (255/1929)		57/1417 (4.02%)	0/53 (0%)	27/439 (6.15%)	11/202 (5.45%)	3/72 (4.17%)	0.127 ^c^	0.1724	N/A
**Measured Vital Signs**									
**Body temperature** **°C** (139/1046)		37.07 ± 0.88 34.4–40.5 (809)	37.07 ± 1.19 35.2–40.0 (26)	36.91 ± 0.87 35.0–40.0 (209)	36.9 ± 0.81 35.2–40.0 (113)	37.1 ± 1.02 36.0–40.0 (28)	0.4907 ^c^	0.0797	N/A
**Heart rate** **beats/minute** (228/1444)		86.41 ± 15.63 48–160 (1045)	84.96 ± 17.79 54–120 (47)	84.01 ± 16.31 50–160 (340)	84.67 ± 19.71 36–170 (181)	85.03 ± 15.78 54–140 (59)	0.923 ^c^	0.1793	N/A
**Respiratory rate breaths/minute** (48/270)		18.35 ± 5.78 12–50 (204)	18.0 ± 4.33 14–28 (12)	18.8 ± 5.68 12–45 (56)	19.92 ± 6.42 12–50 (36)	17.1 ± 4.23 12–24 (10)	0.2538 ^c^	0.5575	N/A
**Systolic blood pressure** (231/1438)		130.72 ± 21.28 60–240 (1040)	126.85 ± 25.82 80–200 (46)	135.24 ± 25.16 50–270 (339)	134.71 ± 25.6 70-205 (185)	133.85 ± 21.83 86-210 (59)	0.0685 ^c^	**0.0102**	**0.008 ^a^** 0.534 ^b^ 0.898 ^c^
**Diastolic blood pressure** (231/1430)		78.55 ± 12.68 40-150 (1037)	77.65 ± 14.27 50-110 (46)	78.11 ± 13.6 40-157 (334)	74.75 ± 15.24 40-120 (185)	78.98 ± 15.16 51-143 (59)	0.2267 ^c^	0.8443	N/A
**SpO_2_ in room air, % (FiO2 = 21%)** (161/1101)		92.84 ± 7.13 48-100 (814)	89.08 ± 11.85 50-99 (37)	89.79 ± 9.29 50-100 (244)	90.1 ± 9.39 50-99 (124)	90.4 ± 5.48 74-98 (43)	0.6345 ^c^	**<0.0001**	**<0.0001 ^a^** **0.019 ^b^** 0.824 ^c^
**Abnormalities Detected during Physical Examination**									
**Crackles** (255/1929)		154/1417 (10.87%)	13/53 (24.53%)	86/439 (19.59%)	56/202 (27.72%)	10/72 (13.89%)	0.7701 ^c^	**<0.0001**	**<0.0001 ^a^** 1.0 ^b^ 0.9737 ^c^
**Wheezing** (255/1929)		94/1417 (6.62%)	7/53 (13.21%)	49/439 (11.16%)	52/202 (25.74%)	17/72 (23.61%)	0.0813 ^c^	**<0.0001**	**0.0079 ^a^** **<0.0001 ^b^** **0.019 ^c^**
**Pulmonary congestion** (255/1929)		184/1417 (12.99%)	19/53 (35.85%)	86/439 (19.59%)	66/202 (32.67%)	12/72 (16.67%)	0.785 ^c^	**0.0025**	**0.0024 ^a^** 1.0 ^b,c^
**Peripheral oedema** (255/1929)		76/1417 (5.36%)	14/53 (26.42%)	46/439 (10.48%)	44/202 (21.78%)	9/72 (12.5%)	0.5947 ^c^	**0.0002**	**0.0011 ^a^** 0.0551 ^b^ 1.0 ^c^

Continuous variables are presented as mean ± SD, range (minimum–maximum), and number of non-missing values. Categorized variables are presented as a number with a percentage. Information about the numbers with valid values is provided in the left column. Abbreviations: SD—standard deviation, CAD—coronary artery disease, OMNIBUS—analysis of variance, N—valid measurements, n—number of patients with parameter above cut-off point, SBP-systolic blood pressure, DBP—diastolic blood pressure, a—low risk vs. medium risk, b—low risk vs. high risk, c—medium risk vs. high risk. Bold text—statistically significant values.

**Table 3:** Applied treatment and procedures.

Variables, Units (N) (HF/Non-HF)	Low-Risk (0–1) n/N		Medium-Risk (2–3) n/N		High-Risk (≥4) n/N		*t*-Test	OMNIBUS *p* Value	*p* Value for Post Hoc Analysis
	Mean ± SD Min–Max (N) or n/N (% of Risk Category)		Mean ± SD Min–Max (N) or n/N (% of Risk Category)		Mean ± SD Min–Max (N) or n/N (% of Risk Category)				
	HF	Non-HF	HF	Non-HF	HF	Non-HF	HF	Non-HF	
Applied Treatment and Procedures									
**The most advanced respiratory support applied during the hospitalization** **no oxygen** (255/1928)							0.4126 ^c^	**<0.0001**	**0.0007 ^a^** **0.0012 ^b^** 0.2081 ^c^
				
				
		741/1417	19/53	183/439	61/202	28/72			
		(52.37%)	(35.85%)	(41.78%)	(30.2%)	(38.89%)			
**low flow oxygen support** (255/1928)									
				
		451/1417	19/53	169/439	90/202	34/72			
		(31.87%)	(35.85%)	(38.58%)	(44.55%)	(47.22%)			
**high flow nasal cannula** **noninvasive ventilation** (255/1928)									
				
				
		65/1417	3/53	36/439	21/202	6/72			
		(4.59%)	(5.66%)	(8.22%)	(10.4%)	(8.33%)			
**invasive ventilation** (255/1928)									
				
		141/1417	8/53	41/439	21/202	1/72			
		(9.96%)	(15.09%)	(9.36%)	(10.4%)	(1.39%)			
**Oxygenation parameters from the period of qualification for advanced respiratory support: SpO_2_, %** (87/544)		90.63 ± 7.88 50–100 (410)	88.33 ± 8.67 72–98 (12)	86.31 ± 9.83 55–99 (121)	84.63 ± 10.31 59–99 (75)	91.08 ± 5.01 81–98 (13)	0.2002 ^c^	**0.0004**	**<0.0001 ^a^** 0.948 ^b^ 0.022 ^c^
**Therapy with catecholamines** (255/1928)		131/1417 (9.24%)	7/53 (13.21%)	38/439 (8.66%)	38/202 (18.81%)	4/72 (5.56%)	0.4532 ^c^	0.5456	N/A
**Coronary revascularization** **or/and an indication for coronary revascularization** (255/1928)		10/1417 (0.71%)	4/53 (7.55%)	8/439 (1.82%)	8/202 (3.96%)	0/72 (0%)	0.2796 ^c^	0.0927	N/A
**Hemodialysis** (255/1928)		46/1417 (3.25%)	5/53 (9.43%)	8/439 (1.82%)	12/202 (5.94%)	0/72 (0%)	0.3601 ^c^	0.1196	N/A

Continuous variables are presented as: mean ± SD, range (minimum–maximum), and number of non-missing values. Categorized variables are presented as: a number with a percentage. Information about the numbers with valid values is provided in the left column. Abbreviations: CAD—coronary artery disease, OMNIBUS—analysis of variance, N–valid measurements, n—number of patients with parameter above cut-off point, SD—standard deviation, N/A—not applicable, a—low risk vs. medium risk, b—low risk vs. high risk, c—medium risk vs. high risk. Bold text–statistically significant values.

**Table 4:** Total and in-hospital all-cause mortality in the C_2_HEST risk strata in diabetes and non-diabetes cohorts.

Variables, Units (N) (HF/Non-HF)	Low-Risk (0–1)		Medium-Risk (2–3)		High-Risk (≥4)		*t*-Test	OMNIBUS *p* Value	*p* Value for Post Hoc Analysis
	n/N (% of Risk Category)		n/N (% of Risk Category)		n/N (% of Risk Category)				
	HF	Non-HF	HF	Non-HF	HF	Non-HF	HF	Non-HF	
All-Cause Mortality Rate									
**In-hospital mortality** (255/1928)		119/1417 (8.4%)	11/53 (20.75%)	99/439 (22.55%)	78/202 (38.61%)	19/72 (26.39%)	0.0235 ^c^	**<0.0001**	**<0.0001 ^a,b^** 1.0 ^c^
**3-month mortality** (255/1928)		202/1417 (14.26%)	23/53 (43.4%)	175/439 (39.86%)	109/202 (53.96%)	38/72 (52.78%)	0.2242 ^c^	**<0.0001**	**<0.0001 ^a,b^** 0.1604 ^c^
**6-month mortality** (220/1270)		214/867 (24.68%)	24/41 (58.54%)	184/343 (53.64%)	117/179 (65.36%)	39/60 (65.0%)	0.5212 ^c^	**<0.0001**	**<0.0001 ^a,b^** 0.4074 ^c^

Continuous variables are presented as mean ± SD, range (minimum–maximum), and number of non-missing values. Categorized variables are presented as a number with a percentage. Information about the numbers with valid values is provided in the left column. Abbreviations: CAD—coronary artery disease, OMNIBUS—analysis of variance, N—valid measurements, n—number of patients with parameter above cut-off point, a—low risk vs. medium risk, b—low risk vs. high risk, c—medium risk vs. high risk; Bold text—statistically significant values.

**Table 5:** The total all-cause death hazard ratios for C_2_HEST risk stratification in the heart failure cohort.

Total Death			
**Overall**	**HR**	**95%CI**	***p* Value**
	1.100	0.968–1.250	0.143
**Risk Strata**			
**Medium-Risk vs. High-Risk**	1.465	0.951–2.259	0.085

**Table 6:** The total all-cause-death Hazard Ratios for C_2_HEST risk stratification in non-HF cohort.

Total Death			
**Overall**	**HR**	**95%CI**	***p* Value**
	1.623	1.518–1.734	<0.0001
**Risk Strata**			
**Low-Risk vs. Medium-Risk**	3.414	2.811–4.148	<0.0001
**Low-Risk vs. High-Risk**	4.953	3.570–6.873	<0.0001

**Table 7:** Associations of individual C_2_HEST score components with mortality in the HF cohort.

	Component	HR	CI Min.	CI Max.	*p* Value
All-cause mortality	Coronary artery disease	1.1759	0.8376	1.6509	0.3492
	COPD	1.3432	0.8127	2.2201	0.2498
	Age > 75	1.4743	1.0561	2.0581	0.0226
	Thyroid disease	0.5794	0.3476	0.9658	0.0363
	Hypertension	0.9133	0.5895	1.4151	0.6849
	HFrEF	NA	NA	NA	NA

Abbreviations: COPD—chronic obstructive pulmonary disease, HFrEF—heart failure with reduce ejection fraction.

**Table 8:** Associations of individual C_2_HEST score components with mortality in non-HF cohort.

	Component	HR	CI Min.	CI Max.	*p* Value
All-cause mortality	Coronary artery disease	1.8775	1.4009	2.5162	<0.0001
	COPD	1.6793	1.0969	2.5707	0.017
	Age > 75	3.2112	2.6317	3.9183	<0.0001
	Thyroid disease	0.8555	0.6201	1.1804	0.3421
	Hypertension	1.3936	1.1383	1.7062	0.0013
	HFrEF	NA	NA	NA	NA

Abbreviations: COPD—chronic obstructive pulmonary disease, HFrEF—heart failure with reduce ejection fraction.

**Table 9:** Clinical non-fatal events and hospitalization outcomes in the C_2_HEST risk strata in heart failure and non-failure cohorts.

Variables, Units (N) (HF/Non-HF)	Low-Risk (0–1)		Medium-Risk (2–3)		High-Risk (≥4)		*t*-Test	OMNIBUS *p* Value	*p* Value for Post Hoc Analysis
	Mean ± SD Min–Max (N) or n/N (% of Risk Category)		Mean ± SD Min–Max (N) or n/N (% of Risk Category)		Mean ± SD Min–Max (N) or n/N (% of Risk Category)				
	HF	Non-HF	HF	Non-HF	HF	Non-HF	HF	Non-HF	
Hospitalization									
**Duration of hospitalization, days** (255/1928)		11.48 ± 13.66 1–131 (1417)	13.34 ± 9.8 1–39 (53)	13.16 ± 14.0 1–124 (439)	16.02 ± 14.62 1–87 (202)	16.25 ± 19.08 1–121 (72)	0.1162 ^c^	**0.0154**	0.072 ^a^ 0.098 ^b^ 0.389 ^c^
**Admission at ICU** (255/1928)		150/1417 (10.59%)	7/53 (13.21%)	31/439 (7.06%)	25/202 (12.38%)	8/72 (2.78%)	1.0 ^c^	**0.0125**	0.1118 ^a^ 0.1589 ^b^ 0.7983 ^c^
**End of hospitalization** **death** (255/1928)		119/1417 (8.4%)	11/53 (20.75%)	99/439 (22.55%)	78/202 (38.61%)	19/72 (26.39%)	0.0606 ^c^	**<0.0001**	**<0.0001 ^a,b^** 0.9339 ^c^
**discharge to home–** **full recovery**		993/1417 (70.08%)	23/53 (43.4%)	197/439 (44.87%)	75/202 (37.13%)	20/72 (38.89%)			
**transfer to another hospital—worsening**		139/1417 (9.81%)	12/53 (22.64%)	85/439 (19.36%)	25/202 (12.38%)	19/72 (26.39%)			
**transfer to another hospital—in recovery**		166/1417 (11.71%)	7/53 (13.21%)	58/439 (13.21%)	24/202 (11.88%)	6/72 (8.33%)			
**Aborted cardiac arrest** (255/1928)		15/1417 (1.06%)	1/53 (1.89%)	2/439 (0.46%)	5/202 (2.48%)	1/72 (1.59%)	1.0 ^c^	0.3613	N/A
**Shock** (255/1928)		108/1417 (7.62%)	8/53 (15.09%)	39/439 (8.66%)	30/202 (14.85%)	3/72 (4.17%)	1.0 ^c^	0.3999	N/A
**Hypovolemic shock** (255/1928)		22/1417 (1.55%)	1/53 (1.89%)	6/439 (1.37%)	5/202 (2.48%)	1/72 (1.39%)	1.0 ^c^	1.0	N/A
**Cardiogenic shock** (255/1928)		7/1417 (0.49%)	3/53 (5.66%)	8/439 (1.82%)	13/202 (6.44%)	1/72 (1.39%)	1.0 ^c^	**0.0196**	**0.036 ^a^** 0.9839 ^b^ 1.0 ^c^
**Septic shock** (255/1928)		88/1417 (6.21%)	4/53 (7.55%)	26/439 (5.92%)	20/202 (9.9%)	2/72 (2.78%)	0.793 ^c^	0.576	N/A
**Venous thromboembolic disease** (255/1928)		83/1417 (5.86%)	5/53 (9.43%)	25/439 (5.69%)	13/202 (6.44%)	2/72 (2.78%)	0.5451 ^c^	0.6649	N/A
**Pulmonary embolism** (255/1928)		78/1417 (5.86%)	4/53 (7.55%)	24/439 (5.69%)	13/202 (6.44%)	2/72 (2.78%)	0.2498 ^c^	0.98	N/A
**Myocardial infarction** (255/1928)		8/1417 (0.56%)	3/53 (5.66%)	7/439 (0.59%)	7/202 (3.47%)	1/72 (1.39%)	0.4379 ^c^	0.078	N/A
**Myocardial injury** (185/989)		113/678 (16.67%)	15/39 (38.46%)	83/266 (31.2%)	70/146 (47.95%)	17/45 (37.78%)	0.3816 ^c^	**<0.0001**	**<0.0001 ^a^** **0.0023 ^b^** 1.0 ^c^
**Acute heart failure** (255/1928)		8/1417 (0.56%)	11/53 (20.75%)	11/439 (2.51%)	42/202 (20.79%)	4/72 (5.56%)	1.0 ^c^	**<0.0001**	**0.004 ^a^** **0.0056 ^b^** 0.7389 ^c^
**Stroke/TIA** (255/1928)		18/1417 (1.27%)	3/53 (5.66%)	16/439 (3.64%)	4/202 (1.98%)	3/72 (4.17%)	0.1591 ^c^	**0.0023**	**0.0099 ^a^** 0.2315 ^b^ 1.0 ^c^
**New cognitive signs and symptoms** (255/1928)		38/1417 (2.68%)	7/53 (13.21%)	44/439 (10.02%)	22/202 (10.89%)	10/72 (13.89%)	0.8183 ^c^	**<0.0001**	**<0.0001 ^a^** **0.0002 ^b^** 0.9175 ^c^
**Pneumonia** (255/1928)		682/1417 (48.13%)	35/53 (66.04%)	270/439 (61.5%)	141/202 (69.8%)	45/72 (62.5%)	0.7184 ^c^	**<0.0001**	**<0.0001 ^a^** 0.0717 ^b^ 1.0 ^c^
**Complete respiratory failure** (60/216)		57/121 (47.11%)	5/10 (50.0%)	41/78 (52.56%)	36/50 (72.0%)	7/17 (41.18%)	0.2632 ^c^	0.6146	N/A
**SIRS** (255/1860)		142/1352 (10.5%)	8/53 (15.09%)	34/436 (7.8%)	27/202 (13.43%)	9/72 (12.5%)	0.9297 ^c^	0.1981	N/A
**Sepsis** (118/766)		9/576 (1.56%)	3/24 (12.5%)	4/159 (2.52%)	7/94 (7.45%)	0/31 (0%)	0.4228 ^c^	**0.0077**	0.3098 ^a^ **0.0256 ^b^** 0.9164 ^c^
**Acute kidney injury** (255/1928)		110/1417 (7.76%)	9/53 (16.98%)	58/439 (13.21%)	47/202 (23.27%)	12/72 (16.67%)	0.4252 ^c^	**0.0003**	**0.0022 ^a^** **0.0409 ^b^** 1.0 ^c^
**Acute liver dysfunction** (239/1735)		30/1256 (2.39%)	5/50 (10.0%)	17/415 (4.1%)	11/189 (5.82%)	3/64 (4.69%)	0.338 ^c^	0.0951	N/A
**Multiple organ dysfunction syndrome** (255/1928)		21/1417 (1.48%)	4/53 (7.55%)	4/439 (0.91%)	8/202 (3.96%)	0/72 (0%)	0.2796 ^c^	0.5482	N/A
**Lactic acidosis** (55/190)		9/105 (8.57%)	0/10 (0%)	5/69 (7.25%)	8/45 (17.78%)	0/16 (0%)	0.3263 ^c^	0.7588	N/A
**Bleedings** (255/1928)		64/1417 (4.52%)	2/53 (3.77%)	23/439 (5.24%)	21/202 (10.4%)	4/72 (5.56%)	0.1802 ^c^	0.6717	N/A

Continuous variables are presented as mean ± SD, range (minimum–maximum), and number of non-missing values. Categorized variables are presented as a number with a percentage. Information about the numbers with valid values is provided in the left column. Abbreviations: CAD—coronary artery disease, OMNIBUS—analysis of variance, N—valid measurements, n—number of patients with parameter above cut-off point, SD—standard deviation, ICU—intensive care unit, TIA —transient ischemic attack, SIRS—systemic inflammatory response syndrome, N/A—not applicable, a—low risk vs. medium risk, b—low risk vs. high risk, c—medium risk vs. high risk. Bold text—statistically significant values.

## Data Availability

The datasets used and/or analyzed during the current study are available from the corresponding author upon reasonable request.

## Supplementary Materials

The following supporting information can be downloaded at: https://www.mdpi.com/article/10.3390/jcm11123495/s1, Table S1: Baseline characteristics of the study cohort-treatment applied before hospitalization; Table S2: Laboratory parameters measured during the hospitalization in the studied cohort. Table S3: Therapies applied during the hospitalization in the studied cohort. Table S4: The Log-rank statistics for matching the C2HEST risk strata for in-hospital mortality in HF cohort. Table S5. The Log-rank statistics for matching the C2HEST risk strata for in-hospital mortality in non-HF cohort.
