# Supplementary material for: Usefulness of C2HEST Score in Predicting Clinical Outcomes of COVID-19 in Heart Failure and Non-Heart-Failure Cohorts
Source: J Clin Med. 2022 Jun 17;11(12):3495. doi: 10.3390/jcm11123495 (PMC9225357; doi:10.3390/jcm11123495)
Supplement: Supplementary file 1 [file jcm-11-03495-s001.zip › jcm-1739862-SI.pdf]

Supplementary Materials

Supplementary Table S1. Baseline characteristics of the study cohort - treatment applied before hospitalization.

Variables, units<br>(N)<br>(HF/non-HF)	Low risk<br>[0-1]<br>n/N<br>(% of risk category)		Medium risk<br>[2-3]<br>n/N<br>(% of risk category)		High risk<br>[≥4]<br>n/N<br>(% of risk category)		*t-test*	OMNIBUS<br>*p value*	*p value*<br>for post-hoc<br>analysis
	HF	Non-HF	HF	Non-HF	HF	Non-HF	HF	Non-HF	
				Treatment applied before hospitalization					
**ACEI**<br>(255/1929)		116/1417<br>(8.19%)	20/53<br>(37.74%)	100/439<br>(22.78%)	84/202<br>(41.58%)	32/72<br>(44.44%)	0.7261 [c]	**<0.0001**	**<0.0001 [a,b]**<br>**0.0005 [c]**
**ARB**<br>(255/1929)		76/1417<br>(5.36%)	1/53<br>(1.89%)	37/439<br>(8.43%)	23/202<br>(11.39%)	7/72<br>(9.72%)	**0.0344 [c]**	**0.0258**	0.0668 [a]<br>0.3458 [b]<br>1.0 [c]
**MRA**<br>(255/1929)		18/1417<br>(1.27%)	12/53<br>(22.64%)	21/439<br>(4.78%)	44/202<br>(21.78%)	5/72<br>(6.94%)	1.0 [c]	**<0.0001**	**<0.0002 [a]**<br>**0.0117 [b]**<br>1.0 [c]
**β-blocker**<br>(255/1929)		197/1417<br>(13.9%)	29/53<br>(54.72%)	150/439<br>(34.17%)	124/202<br>(61.39%)	33/72<br>(45.83%)	0.4687 [c]	**<0.0001**	**<0.0001[a,b]**<br>0.2248 [c]
**Digitalis glycoside**<br>(255/1929)		3/1417<br>(0.21%)	2/53<br>(3.77%)	5/439<br>(0.91%)	8/202<br>(3.96%)	2/72<br>(2.78%)	1.0 [c]	**<0.0001**	0.1753 [a]<br>0.0629 [b]<br>0.605 [c]
**Calcium channel blocker (non-dihydropiridines)**<br>(255/1929)		11/1417<br>(0.78%)	2/53<br>(3.77%)	11/439<br>(2.51%)	9/202<br>(4.46%)	5/72<br>(6.94%)	1.0 [c]	**<0.0001**	**0.026 [a]**<br>**0.002 [b]**<br>0.1807 [c]
**Calcium channel blocker (dihydropiridines)**<br>(255/1929)		103/1417<br>(7.27%)	6/53<br>(11.32%)	78/439<br>(17.77%)	59/202<br>(29.21%)	15/72<br>(20.83%)	**0.0131 [c]**	**<0.0001**	**<0.0001[a]**<br>**0.0003 [b]**<br>1.0 [c]
**α-adrenergic blocker**<br>(255/1929)		45/1417<br>(3.18%)	6/53<br>(11.32%)	28/439<br>(6.38%)	33/202<br>(16.34%)	6/72<br>(8.33%)	0.4911 [c]	**0.0022**	**0.0135 [a]**<br>0.0993 [b]<br>1.0 [c]
**Thiazide or thiazide-like diuretic**<br>(255/1929)		68/1417<br>(4.8%)	5/53<br>(9.43%)	42/439<br>(9.57%)	23/202<br>(11.39%)	12/72<br>(16.67%)	0.8746 [c]	**<0.0001**	**0.0014 [a]**<br>**0.0008[b]**<br>0.2842[c]
**Loop diuretic**<br>(255/1929)		39/1417<br>(2.75%)	27/53<br>(50.94%)	38/439<br>(8.66%)	73/202<br>(36.14%)	8/72<br>(11.11%)	0.0708 [c]	**<0.0001**	**<0.0001[a]**<br>**0.0042 [b]**<br>1.0 [c]
**Statin**<br>(255/1929)		103/1417<br>(7.27%)	20/53<br>(37.74%)	101/439<br>(23.01%)	103/202<br>(50.99%)	23/72<br>(31.94%)	0.1178 [c]	**<0.0001**	**<0.0001[a,b]**<br>0.4076 [c]
**Acetylsalicylic acid**<br>(255/1929)		81/1417<br>(5.72%)	14/53<br>(26.42%)	81/439<br>(18.45%)	62/202<br>(30.69%)	20/72<br>(27.78%)	0.6619 [c]	**<0.0001**	**<0.0001[a,b]**<br>0.2775 [c]
**LMWH**<br>(255/1929)		74/1417<br>(5.22%)	9/53<br>(16.98%)	32/439<br>(7.29%)	19/202<br>(9.41%)	7/72<br>(9.72%)	0.1858 [c]	0.0883	N/A
**VKA**<br>(255/1929)		10/1417<br>(0.71%)	2/53<br>(3.77%)	12/439<br>(2.73%)	20/202<br>(9.9%)	3/72<br>(4.17%)	0.2684 [c]	**0.0006**	**0.0049 [a]**<br>0.0656 [b]<br>1.0 [c]
**NOAC**<br>(255/1929)		18/1417<br>(1.27%)	8/53<br>(15.09%)	29/439<br>(6.61%)	47/202<br>(23.27%)	5/72<br>(6.94%)	0.2714 [c]	**<0.0001**	**<0.0001[a]**<br>**0.0117 [b]**<br>1.0 [c]
**Insulin**<br>(255/1929)		62/1417<br>(4.38%)	6/53<br>(11.32%)	23/439<br>(5.24%)	33/202<br>(16.34%)	7/72<br>(9.72%)	0.4911 [c]	0.1008	N/A
**Metformin**<br>(255/1929)		104/1417<br>(7.34%)	7/53<br>(13.21%)	60/439<br>(13.67%)	45/202<br>(22.28%)	6/72<br>(8.33%)	0.2051 [c]	**0.0002**	**0.0002 [a]**<br>1.0 [b]<br>0.8656 [c]
**SGLT2 inhibitor**<br>(255/1929)		11/1417<br>(0.78%)	3/53<br>(5.66%)	4/439<br>(0.91%)	5/202<br>(3.96%)	1/72<br>(1.39%)	0.7026 [c]	0.5035	N/A
**Oral antidiabetics other than SGLT2 inhibitor and metformin**<br>(255/1929)		27/1417<br>(1.91%)	5/53<br>(9.43%)	29/439<br>(6.61%)	25/202<br>(12.38%)	3/72<br>(4.17%)	0.7247 [c]	**<0.0001**	**<0.0001 [a]**<br>0.5216 [b]<br>1.0 [c]
**Proton pump inhibitor**<br>(255/1929)		89/1417<br>(6.28%)	15/53<br>(28.3%)	60/439<br>(13.67%)	51/202<br>(%)	15/72<br>(20.83%)	0.4383 [c]	**<0.0001**	**<0.0001[a,b]**<br>0.473 [c]
**Oral corticosteroid**<br>(255/1929)		62/1417<br>(4.38%)	5/53<br>(9.43%)	19/439<br>(4.33%)	4/202<br>(1.98%)	2/72<br>(2.78%)	**0.0209 [c]**	0.9273	N/A
**Immunosuppression other than oral corticosteroid**<br>(255/1929)		49/1417<br>(3.46%)	6/53<br>(11.32%)	16/439<br>(3.64%)	2/202<br>(0.99%)	0/72<br>(0%)	**0.0013 [c]**	0.2908	N/A

Categorized variables are presented as: a number with a percentage. Information about the numbers with valid values is provided in the left column; Abbreviations: CAD – coronary artery disease, OMNIBUS - analysis of variance, N-valid measurements. n - number of patients with parameter above cut-off point, ACEI - angiotensin-converting-enzyme inhibitors, ARBs- angiotensin receptor blockers, MRAs - mineralocorticoid receptor antagonists, LMWH –low molecular weight heparin, VKA- vitamin K antagonists, NOAC - novel oral anticoagulants, SGLT2 inhibitors – sodium glucose co-transporter-2 inhibitors. N/A – non-applicable. a – low risk vs. medium risk, b – low risk vs. high risk, c – medium risk vs. high risk. Bold text- statistically significant values.

Supplementary Table S2. Laboratory parameters measured during the hospitalization in the studied cohort

Parameter (N) (HF/ Non-HF)	Time of assessment	Units	**Low risk [0-1]**<br>mean±SD<br>min-max<br>(N) or n/N<br>(% of risk category)		**Medium risk [2-3]**<br>mean±SD<br>min-max<br>(N) or n/N<br>(% of risk category)		**High risk [≥4]**<br>mean±SD<br>min-max<br>(N) or n/N<br>(% of risk category)		***t-test***	**OMNIBUS *p-value***	***p-value* for post-hoc analysis**
			**HF**	**Non-HF**	**HF**	**Non-HF**	**HF**	**Non-HF**	**HF**	**Non-HF**	
**Complete Blood Count (CBC)**											
**Leucocytes** (251/1798)	**On admission**	x10^3/µl		8.98±12.34<br>0.5-304.02<br>(1301)	9.93.±6.08<br>3.47-35.47<br>(53)	9.28±11.99<br>0.51-215.97<br>(427)	9.53±8.86<br>1.19-99.73<br>(198)	8.63±4.6<br>1.36-27.31<br>(70)	0.7038^{c}	0.7166	**N/A**
**Haemoglobin** (251/1798)	**On admission**	g/dl		13.27±2.15<br>3.9-20.3<br>(1301)	12.55±2.18<br>7.9-17.6<br>(53)	12.57±2.33<br>4.5-18.9<br>(427)	11.84±2.47<br>5.3-18.8<br>(198)	12.27±2.5<br>7.1-17.9<br>(70)	**0.**043^{c}	**<0.0001**	**<0.0001^{a}**<br>**0.005^{b}**<br>0.623^{c}
**Platelets** (251/1798)	**On admission**	x10^3/µl		235.64<br>±108.51<br>0-1356.0<br>(1301)	233.49<br>±109.2<br>106.0-740.0<br>(53)	229.65<br>±114.59<br>3.0-735.0<br>(427)	211.28±91.34<br>15.0-578<br>(198)	232.5±97.89<br>8-502<br>(70)	0.1784^{c}	0.6323	N/A
**Acid-base balance in the arterial blood gas**											
**PH** (60/216)	**On admission**			7.43±0.08<br>7.04-7.58<br>(121)	7.43±0.04<br>7.35-7.52<br>(10)	7.43±0.07<br>7.1-7.54<br>(78)	7.4±0.08<br>7.09-7.54<br>(50)	7.43±0.05<br>7.33-7.53<br>(17)	0.1081^{c}	0.989	N/A
**PaO_2** (60/216)	**On admission**	mmHg		72.25±27.29<br>12.8-100.0<br>(121)	83.22<br>±52.76<br>33.4-100.0<br>(10)	75.44±47.23<br>28.3-100.0<br>(78)	67.06±30.74<br>23.7-100.0<br>(50)	80.99±43.36<br>45.1-100.0<br>(17)	0.3702^{c}	0.6546	N/A
**$PaCO_2$** (60/216)	**On admission**	mmHg		38.02±10.27<br>20.2-82.4<br>(121)	38.89<br>±10.59<br>28.7-59.8<br>(10)	36.37±9.29<br>20.9-79.4<br>(78)	39.23±11.87<br>19.7-88.4<br>(50)	36.94±7.57<br>25.5-61.0<br>(17)	0.9298^{c}	0.5064	N/A
**HCO3 standard** (60/212)	**On admission**	mmol/l		24.91±3.76<br>12.1-32.9<br>(120)	25.55±3.1<br>21.8-32.4<br>(10)	24.21±4.31<br>14.3-39.5<br>(75)	23.92±4.56<br>13.5-25.55<br>(50)	24.84±4.68<br>18.4-36.7<br>(17)	0.1829^{c}	0.5193	N/A
**BE** (21/87)	**On admission**	mmol/l		0.93±4.77<br>[-]15.7-10.5<br>(41)	3.48±4.6<br>[-]1.4-9.7<br>(4)	1.53±5.39<br>[-]12.5-15.7<br>(39)	1.46±4.81<br>[-]3.7-14.6<br>(17)	3.44±6.14<br>[-]7.4-13.2<br>(7)	0.4731^{c}	0.5812	N/A
**Lactates** (55/190)	**On admission**	mmol/l		2.45±1.58<br>0.7-12.8<br>(105)	2.12±0.91<br>0.6-3.7<br>(10)	2.13±1.05<br>0.5-6.4<br>(69)	2.59±1.86<br>0.8-12.0<br>(45)	2.36±1.49<br>0.8-6.0<br>(16)	0.2483^{c}	0.2779	N/A
**Electrolytes, inflammatory and iron biomarkers**											
**Na** (250/1781)	**On admission**	mmol/l		138.26±4.36<br>106.0-159.0 (1288)	137.33<br>±4.24<br>126-147<br>(52)	137.79±7.26<br>101.0-175.0<br>(423)	138.27±6.91<br>108-174<br>(198)	136.96±6.53<br>112-154<br>(70)	0.2206^{c}	0.1307	N/A
**K** (250/1788)	**On admission**	mmol/l		4.06±0.58<br>2.0-7.5<br>(1293)	4.28±0.71<br>3.0±7.03<br>(52)	4.12±0.7<br>2.4-6.8<br>(425)	4.36±0.87<br>2.53-8.7<br>(198)	4.1±0.62<br>2.85-5.9<br>(70)	0.5136^{c}	0.3063	N/A
**CRP** (251/1768)	**On admission**	mg/l		76.46±84.49<br>0.13-531.58<br>(1274)	78.97<br>±96.28<br>1.57-487.38<br>(53)	83.72±85.45<br>0.29-538.55<br>(424)	73.94±78.82<br>0.4-390.94<br>(198)	83.79±87.7<br>0.4-365.22<br>(70)	0.7270^{c}	0.2794	N/A
	**On discharge**			48.26±79.16<br>0.13-496.98<br>(1274)	61.34<br>±78.66<br>0.61-198.0<br>(53)	74.01±96.26<br>0.22-538.55<br>(424)	73.09±84.93<br>0.42-390.94<br>(198)	78.23±92.54<br>0.4-431.9<br>(70)	0.3452^{c}	**<0.0001**	**<0.0001^{a}**<br>**0.026^{b}**<br>0.934^{c}
**Procalcitonin** (205/1269)	**On admission**	ng/ml		0.84±4.48<br>0.01-61.28<br>(918)	2.21<br>±11.06<br>0.01-72.61<br>(43)	1.78±12.17<br>0.01-196.04<br>(301)	1.61±6.85<br>0.01-60.77<br>(162)	1.04±2.31<br>0.01-12.99<br>(50)	0.7369^{c}	0.3903	N/A

**IL-6**<br>(70/632)	**On admission**	pg/ml	61.29±424.31<br>2-9099<br>(480)	31.69<br>±30.99<br>2.33-108<br>(14)	44.46±66.18<br>2-499<br>(129)	71.7±100.55<br>2.0-421.0<br>(56)	45.32±83.56<br>2.0-373<br>(23)	**0.0137**[c]	0.7106	N/A
**D-dimer**<br>(193/1386)	**On admission**	µg/ml	3.73±12.19<br>0.15-132.82<br>(1002)	7.65<br>±21.95<br>0.33-127.24<br>(42)	6.35±16.02<br>0.2-107.65<br>(331)	5.53±16.98<br>0.22-128.0<br>(151)	5.03±18.14<br>0.27-128<br>(53)	0.5642[c]	**0.0259**	**0.018**[a]<br>0.864[b]<br>0.873[c]
**INR**<br>(233/1691)	**On admission**		1.14±0.48<br>0.82-15.2<br>(1227)	1.41±0.64<br>0.89-4.33<br>(47)	1.24±0.59<br>0.87-7.8<br>(398)	1.98±2.8<br>0.9-21.1<br>(186)	1.22±0.36<br>0.89-2.79<br>(66)	**0.0125**[c]	**0.0026**	**0.004**[a]<br>0.172[b]<br>0.908[c]
**aPTT**<br>(227/1640)	**On admission**	>60 s	28/1191<br>(2.35%)	0/47<br>(0%)	7/384<br>(1.82%)	11/180<br>(6.11%)	0/65<br>(0%)	0.126[c]	0.5682	N/A
**Fibrinogen**<br>(61/359)	**On admission**	g/dl	4.89±1.85<br>0.35-10.0<br>(285)	4.65±2.11<br>1.74-9.2<br>(15)	4.74±1.77<br>0.35-9.04<br>(66)	4.42±1.42<br>1.78-8.11<br>(46)	5.08±2.75<br>2.26-9.1<br>(8)	0.6998[c]	0.8129	N/A
					**Biochemistry**					
**Glucose**<br>(234/1525)	**On admission**	mg/dl	134.84±74.9<br>28.0-933.0<br>(1063)	164.5<br>±91.12<br>61-397<br>(50)	149.45<br>±92.05<br>47.0-1026.0<br>(399)	153.36<br>±102.33<br>37-1064<br>(184)	142.78<br>±86.59<br>70.0-685.0<br>(63)	0.4576[c]	**0.0182**	**0.013**[a]<br>0.757[b]<br>0.84[c]
**Glycated hemoglobin (HbA1c)**<br>(65/198))	**On admission**	%	7.61±2.31<br>4.2-14.9<br>(127)	8.01±1.83<br>4.8-12.9<br>(14)	7.43±2.26<br>4.9-16.6<br>(61)	7.2±1.58<br>5.1-11.9<br>(51)	7.61±2.6<br>5.1-13.7<br>(10)	0.1447[c]	0.8832	N/A
**Urea**<br>(241/1617)	**On admission**	mg/dl	42.84±35.95<br>5.0-307.0<br>(1145)	69.22<br>±48.86<br>10-271<br>(51)	63.84<br>±49.58<br>8.0-353.0<br>(404)	82.76±55.69<br>17-369<br>(190)	61.65<br>±44.03<br>12.0-249.0<br>(68)	0.0917[c]	**<0.0001**	**<0.0001**[a]<br>**0.003**[b]<br>0.926[c]
**Creatinine**<br>(251/1711)	**On admission**	mg/dl	1.15±1.18<br>0.26-14.87<br>(1216)	1.67±1.8<br>0.58-12.66<br>(53)	1.42±1.23<br>0.48-9.56<br>(425)	1.92±1.61<br>0.49-11.3<br>(198)	1.48±1.46<br>0.44-9.49<br>(70)	0.3601	**0.0002**	**0.0002**[a]<br>0.158[b]<br>0.952[c]
	**On discharge**		1.08±1.05<br>0.26-14.87<br>(1216)	1.65±1.8<br>0.51-12.35<br>(53)	1.41±1.29<br>0.43-9.09<br>(425)	1.74±1.45<br>0.43-9.27<br>(198)	1.44±1.34<br>0.43-9.2<br>(70)	0.749[c]	**<0.0001**	**<0.0001**[a]<br>0.074[b]<br>0.983[c]
**eGFR**<br>(251/1706)	**On admission**	ml/min/1,73 m^2	85.01±34.32<br>0-433.0<br>(1211)	63.43<br>±33.79<br>4.0-149.0<br>(53)	61.83<br>±28.12<br>4-137.0<br>(425)	49.96±29.16<br>5.0-180.0<br>(198)	60.93<br>±29.99<br>5.0-145.0<br>(70)	**0.0098**[c]	**<0.0001**	**<0.0001**[a,b]<br>0.97[c]
	**On discharge**		89.34±34.73<br>0-433.0<br>(1211)	65.25<br>±34.12<br>4.0-172.0<br>(53)	65.46<br>±30.59<br>4.0-208<br>(425)	57.26±34.39<br>5.0-209.0<br>(198)	61.71<br>±28.98<br>5.0-148.0<br>(70)	0.1348[c]	**<0.0001**	**<0.0001**[a,b]<br>0.582[c]
**Total protein**<br>(119/487)	**On admission**	g/l	6.06±0.84<br>3.5-8.2<br>(331)	6.08±0.8<br>4.6-7.9<br>(26)	5.91±0.94<br>3.6-9.5<br>(126)	5.72±0.89<br>3.3-8.2<br>(93)	5.82±0.83<br>4.6-8.1<br>(30)	**0.0491**[c]	0.1225	N/A
**Albumin**<br>(129/535)	**On admission**	g/l	3.16±0.6<br>1.5-5.1<br>(374)	3.26±0.57<br>2.1-4.3<br>(29)	3.06±0.55<br>1.1-4.4<br>(131)	2.97±0.6<br>1.5-4.9<br>(100)	2.94±0.63<br>0.7-4.2<br>(30)	**0.0216**[c]	0.0645	N/A
**AST**<br>(202/1240)	**On admission**	IU/L	60.13±114.0<br>5.0-2405.0<br>(883)	36.49<br>±31.12<br>7-161.0<br>(43)	70.74<br>±275.39<br>8.0-4776.0<br>(304)	78.33<br>±221.33<br>10-2518<br>(159)	109.57<br>±526.35<br>8.0-3866.0<br>(53)	**0.0225**[c]	0.6463	N/A
**ALT**<br>(216/1373)	**On admission**	IU/L	54.93±92.84<br>4.0-1411.0<br>(972)	40.56±45.1<br>4.0-222.0<br>(48)	50.22<br>±202.18<br>6.0-3700.0<br>(343)	53.02<br>±130.65<br>6.0-1315<br>(168)	48.03<br>±176.14<br>5.0-1361.0<br>(58)	0.3003[c]	0.8821	N/A
**Bilirubin**<br>(196/1211)	**On admission**	mg/dl	0.84±1.44<br>0.1-19.1<br>(852)	1.02±0.82<br>0.2-4.2<br>(44)	0.8±0.72<br>0.2-9.2<br>(308)	0.94±0.78<br>0.3-6.6<br>(152)	0.68±0.33<br>0.1-1.8<br>(51)	0.5499[c]	**0.0453**	0.829[a]<br>0.051[b]<br>0.124[c]
**LDH**<br>(168/1064)	**On admission**	U/L	429.98<br>±378.19<br>50.0-7100.0<br>(776)	333.02<br>±184.29<br>136.0-894.0<br>(41)	401.02<br>±203.38<br>44.0-1353.0<br>(245)	471.96<br>±841.12<br>106.0-9505.0<br>(127)	350.81<br>±171.17<br>71-798<br>(43)	0.0844[c]	**0.0241**	0.272[a]<br>**0.024**[b]<br>0.205[c]

**Cardiac biomarkers**										
**BNP**<br>(96/263)	**On admissio n**	pg/ml	220.04<br>±638.68<br>1.7-69224.2<br>(161)	1322.53<br>±1408.65<br>17.5-4890.6<br>(18)	251.59<br>±415.69<br>3.0-2712.8<br>(82)	1099.28<br>±2113.49<br>5.9-13368.4<br>(78)	417.46<br>±1733.<br>8<br>22.3-<br>7954.2<br>(20)	0.588<br>7[c]	0.435	N/A
**NT-proBNP**<br>(109/270)	**On admissio n**	ng/ml	1888.76<br>± 7779.04<br>12.0-<br>70000.0<br>(172)	13551.51<br>±16990.58<br>18.2<br>--70000<br>(25)	6974.79<br>±135552.1<br>49.6<br>-70000.0<br>(84)	13905.75<br>±18671.84<br>211.4-70000<br>(84)	15417.<br>6<br>±2198<br>0.0<br>119.6<br>-<br>70000.<br>0<br>(44)	0.929<br>2[c]	**0.0025**	**0.005** [a]<br>0.092[b]<br>0.369 [c]
	**On admissio n**	pg/ml	136.64<br>±807.93<br>0-11758.2<br>(678)	757.13<br>±2739.4<br>1.0<br>-16175.7<br>(39)	1845.89<br>±12315.4<br>1.9-<br>12593.0<br>(266)	914.5<br>±2973.73<br>3.3-21022.9<br>(146)	265.96<br>±698.0<br>6<br>4.0-<br>3342.7<br>(45)	0.755<br>4[c]	**0.0454**	0.066 [a]<br>0.464 [b]<br>0.101 [c]
**Troponin I**<br>(185/989)	<3-fold upper range<br>K 46.8<br>M 102.6		565/678<br>(83.33%)	24/39<br>(61.54%)	183/266<br>(68.8%)	79/146<br>(52.5%)	28/45<br>(62.22<br>%)	0.381<br>6[c]	**<0.0001**	**<0.000 1[a]**<br>**0.0023** [b]<br>1.0 [c]
	> 3-fold upper range<br>K 46.8<br>M 102.6		113/678<br>(16.67%)	15/39<br>(38.46%)	83/266<br>(31.2%)	70/146<br>(47.95%)	17/45<br>(37.78<br>%)			
	**On discharge**	pg/ml	116.86<br>±827.21<br>0.2-12391.6<br>(678)	4960.87<br>±27937.97<br>0.8-174652.6<br>(39)	1410.27<br>±9443.85<br>1.6-<br>109360.0<br>(261)	763.07<br>±3149.13<br>3.9-29828.3<br>(146)	338.09<br>±811.2<br>1.8-<br>3848.7<br>(45)	0.354<br>8[c]	**0.0215**	0.068 [a]<br>0.19 [b]<br>0.167 [c]
**LDL-cholesterol**<br>(86/636)	**On admissio n**	mg/dl	100.07±50.8<br>8<br>6.0-510.0<br>(232)	76.33<br>±33.07<br>25-137<br>(18)	88.91<br>±41.54<br>17.0-230.0<br>(111)	70.81±42.98<br>6.0-210.0<br>(68)	88.55<br>±37.64<br>27.0-<br>187.0<br>(20)	0.559<br>7[c]	0.0832	N/A
**HDL-cholesterol**<br>(84/367)	**On admissio n**	mg/dl	39.98±16.07<br>2.0-120.0<br>(236)	39.33<br>±11.41<br>26-60<br>(18)	40.32<br>±15.96<br>7.0-110.0<br>(111)	36.88±14.61<br>8.0-79.0<br>(66)	38.3±1<br>6.53<br>22.0-<br>79.0<br>(20)	0.453<br>3[c]	0.8819	N/A
**Triglycerid es**<br>(117/523)	**On admissio n**	mg/dl	179.06±124.<br>09<br>40.0-1100.0<br>(359)	144.33<br>±64.03<br>58-328<br>(24)	144.49<br>±101.11<br>48.0-595.0<br>(140)	128.11±66.0<br>46.0-413.0<br>(93)	135.38<br>±40.85<br>51.0-<br>223.0<br>(24)	0.278<br>6[c]	**0.0001**	**0.004** [a]<br>**0.0004**[b]<br>0.727 [c]
**Hormones**										
**25-hydroxy-vitamin D**<br>(61/413)	**On admissio n**	ng/ml	24.71±17.57<br>3.5-146.1<br>(305)	16.47<br>±14.15<br>49.0-62.8<br>(16)	26.22<br>±16.52<br>3.5-77.7<br>(92)	18.87±15.21<br>3.5-63.5<br>(45)	19.99<br>±13.67<br>3.5-<br>46.4<br>(16)	0.572<br>5[c]	0.2809	N/A
**TSH**<br>(136/684)	**On admissio n**	mIU/l	1.35±1.54<br>0-18.6<br>(441)	1.69±1.68<br>0.08-8.28<br>(32)	1.53±2.56<br>0.01-28.81<br>(200)	2.04±3.94<br>0-38.24<br>(104)	2.53±4<br>.04<br>0.06-<br>22.61<br>(43)	0.469<br>3[c]	0.1192	N/A

Continuous variables are presented as: mean ± SD. range (minimum -maximum) and number of non-missing values. Categorized variables are presented as: a number with a percentage. Information about the numbers with valid values is provided in the left column; Abbreviations: CAD – coronary artery disease, OMNIBUS - analysis of variance, N-valid measurements, n - number of patients with parameter above cut-off point, SD - standard deviation, N/A – non-applicable. a – low risk vs. medium risk, b – low risk vs. high risk, c – medium risk vs. high risk. Bold text- statistically significant values.

Supplementary Table S3. Therapies applied during the hospitalization in the studied cohort.

Variables, units<br>(N)<br>(HF/non-HF)	Low risk<br>[2-3]<br>n/N<br>(% of risk category)		Medium risk<br>[2-3]<br>n/N<br>(% of risk category)		High risk<br>[>4]<br>n/N<br>(% of risk category)		*t-test*	OMNIBUS<br>*p-value*	*p-value*<br>for post-hoc analysis
	HF	Non-HF	HF	Non-HF	HF	Non-HF	HF	Non- HF	
**Applied treatment and procedures**									
**Systemic corticosteroid**<br>(255/1928)		708/1417<br>(49.86%)	27/53<br>(50.94%)	219/439<br>(49.89%)	102/202<br>(50.5%))	40/72<br>(55.56%)	1.0 [c]	0.6462	N/A
**Convalescent plasma**<br>(255/1928)		167/1417<br>(11.79%)	6/53<br>(11.32%)	35/439<br>(7.97%)	27/202<br>(13.37%)	4/72<br>(5.56.%)	0.869 [c]	**0.0278**	0.0938 [a]<br>0.4599 [b]<br>1.0 [c]
**Tocilizumab**<br>(255/1928)		22/1417<br>(1.55%)	0/53<br>(0%)	2/439<br>(0.46%)	1/202<br>(0.5%)	0/72<br>(0%)	1.0 [c]	0.144	N/A
**Remdesivir**<br>(255/1928)		236/1417<br>(16.65%)	10/53<br>(18.87%)	62/439<br>(14.12%)	28/202<br>(13.86%)	7/72<br>(9.72%)	0.4875 [c]	0.1571	N/A
**Antibiotic**<br>(255/1928)		746/1417<br>(52.65%)	32/53<br>(60.38%)	271/439<br>(61.73%)	136/202<br>(67.33%)	55/72<br>(76.39%)	0.4313 [c]	**<0.0001**	**0.003** [a]<br>**0.0004** [b]

	0.0703 [c]

Categorized variables are presented as: a number with a percentage. Information about the numbers with valid values is provided in the left column; Abbreviations: CAD – coronary artery disease, OMNIBUS - analysis of variance, N-valid measurements, n - number of patients with parameter above cut-off point, SD - standard deviation, N/A – non-applicable, a – low risk vs. medium risk, b – low risk vs. high risk, c – medium risk vs. high risk. Bold text- statistically significant values.

Supplementary Table S4. The Log-rank statistics for matching the C_2HEST risk strata for in-hospital mortality in HF cohort.

	h2	h3	h4	h5	h6	h7	h8
m1	NA	0.493	1.2036	0.9175	0.8446	0.9471	NA
m2		0.493	1.2036	0.9175	0.8446	0.9471	NA
m3			1.4486	0.9217	0.8664	1.0936	0.4930
m4				1.525	1.706	2.0879	1.2036
m5					1.0258	1.4219	0.9175
m6						1.1337	0.8446
m7							0.9471

Abbreviations: m-medium. h-high.

Supplementary Table S5. The Log-rank statistics for matching the C_2HEST risk strata for in-hospital mortality in non-HF cohort.

	h2	h3	h4	h5	h6	h7	h8
m1	206.8408	166.9896	147.0314	123.2135	10.9153	10.9153	10.9153
m2		195.1092	200.8348	195.5155	13.8904	13.8904	13.8904
m3			118.4059	114.7761	10.6046	10.6046	10.6046
m4				53.3791	7.2096	7.2096	7.2096
m5					2.3469	2.3469	2.3469

Abbreviations: m-medium. h-high.
